# Acylhydrazones as Antifungal Agents Targeting the Synthesis of Fungal Sphingolipids

**DOI:** 10.1128/AAC.00156-18

**Published:** 2018-04-26

**Authors:** Cristina Lazzarini, Krupanandan Haranahalli, Robert Rieger, Hari Krishna Ananthula, Pankaj B. Desai, Alan Ashbaugh, Michael J. Linke, Melanie T. Cushion, Bela Ruzsicska, John Haley, Iwao Ojima, Maurizio Del Poeta

**Affiliations:** a Department of Molecular Genetics and Microbiology, Stony Brook University, Stony Brook, New York, USA; b Veterans Administration Medical Center, Northport, New York, USA; c Institute of Chemical Biology and Drug Discovery, Stony Brook University, Stony Brook, New York, USA; d Proteomics Center, Stony Brook University, Stony Brook, New York, USA; e Department of Pharmaceutical Sciences, University of Cincinnati, Cincinnati, Ohio, USA; f Department of Veterans Affairs Medical Center, Cincinnati, Ohio, USA; g University of Cincinnati College of Medicine, Cincinnati, Ohio, USA; h Department of Chemistry, Stony Brook University, Stony Brook, New York, USA; i Division of Infectious Diseases, School of Medicine, Stony Brook University, Stony Brook, New York, USA

**Keywords:** acylhydrazones, antifungals, Aspergillus fumigatus, Candida albicans, Cryptococcus neoformans, fungal infection, fungi, sphingolipids, infectious disease, pharmacokinetics

## Abstract

The incidence of invasive fungal infections has risen dramatically in recent decades. Current antifungal drugs are either toxic, likely to interact with other drugs, have a narrow spectrum of activity, or induce fungal resistance. Hence, there is a great need for new antifungals, possibly with novel mechanisms of action. Previously our group reported an acylhydrazone called BHBM that targeted the sphingolipid pathway and showed strong antifungal activity against several fungi. In this study, we screened 19 derivatives of BHBM. Three out of 19 derivatives were highly active against Cryptococcus neoformans *in vitro* and had low toxicity in mammalian cells. In particular, one of them, called D13, had a high selectivity index and showed better activity in an animal model of cryptococcosis, candidiasis, and pulmonary aspergillosis. D13 also displayed suitable pharmacokinetic properties and was able to pass through the blood-brain barrier. These results suggest that acylhydrazones are promising molecules for the research and development of new antifungal agents.

## INTRODUCTION

In the past few decades, the incidence of invasive mycoses has increased dramatically principally due to the increase in the population susceptible for these infections, the emergence of new fungal species, and substantial progress in the diagnosis of these infections (1, 2). It was recently estimated that more than 300 million people are diagnosed with serious fungal infections, caused mainly by Cryptococcus, Candida, Aspergillus, and Pneumocystis (3), with approximately 1.5 to 2 million deaths occurring annually as a result of these invasive fungal infections (4). Individuals at high risk include but are not limited to immunocompromised subjects, such as HIV-positive, organ transplant, pediatric, geriatric, and cancer patients and other subjects undergoing immunosuppressive therapy for various reasons (5–8).

The CDC estimates that more than 1 million new cases per year of cryptococcosis will occur worldwide in patients with AIDS, and 600,000 will die from the infection. This is a drastic increase, considering that prior to the mid-1950s, fewer than 300 cryptococcosis cases had been reported in the medical literature (9). Certain medical devices, such as catheters, provide the port of entry to fungi that colonize the skin and mucosa. As a result, disseminated candidiasis is the 4th most common hospital-acquired sepsis, with >120,000 deaths/year (10–12). Another invasive fungal infection, disseminated aspergillosis, is steadily increasing in immunocompromised patients (13–16), with a mortality rate of 450,000/year. Aspergillus spp. are also responsible for severe asthma by fungal sensitization (SAFS), accounting for 100,000 deaths annually. Pneumocystis spp. are a group of host-specific opportunistic fungi that reside in the lungs of humans and animals in nature. The organism is named P. jirovecii in humans, P. carinii in rats, and P. murina in mice. Pneumocystis pneumonia (PCP) remains the most prevalent opportunistic infection in patients infected with HIV. Reports on mortality rates for PCP are variable, ranging from 13% to as high as 80%, which even at the lowest rate results in more than 52,000 deaths per year (17). PCP is also prevalent in other patient groups, notably patients chronically immune suppressed due to solid-organ transplantation or due to chemotherapy for cancer or autoimmune disease. In addition to being the cause of PCP in immunocompromised hosts, P. jirovecii is also a frequent colonizer of the respiratory tract in immunocompetent individuals with other underlying pulmonary diseases, such as chronic obstructive pulmonary disease (COPD), in which it initiates a deleterious inflammatory reaction (18).

Invasive fungal infections are also associated with a prolonged hospital stay, resulting in an increase in hospital cost (19). Current major classes of antifungal drugs include azoles (e.g., fluconazole), polyenes (e.g., amphotericin B), and echinocandins (e.g., caspofungin) (20). These drugs come with their own range of challenges, such as nephrotoxicity, drug-drug interactions, narrow spectrum of activity, and resistance (21–24). For these reasons, there is an urgent need for a new class of antifungal drugs targeting a different fungal pathway(s).

In our previous studies, we showed that one of the new targets for antifungal drug development could be the sphingolipid pathway (25). In particular, we showed the identification and characterization of a synthetic drug called (E)-N′-(3-bromo-6-hydroxybenzylidene)-2-methylbenzohydrazide (BHBM), targeting the synthesis of the fungal and not mammalian sphingolipid glucosylceramide (GlcCer), and its efficacy *in vitro* and *in vivo* against a series of pathogenic fungi. GlcCer is very important for the pathogenicity of C. neoformans (26–28) and other fungi. In C. albicans, mutant strains that cannot make GlcCer showed normal morphological switching and proliferation but proved to be less virulent in animal models (29). In Aspergillus fumigatus, inhibition of GlcCer affects sporulation and hyphal growth, leading to attenuated virulence (30).

In a continued effort to identify potent and safe antifungal compounds with suitable pharmacokinetic properties compared to those of the parent BHBM compound, we screened 19 derivatives of BHBM for antifungal activity, toxicity, and other drug properties in this study.

## RESULTS

### Screening of derivatives.

**FIG 1:**
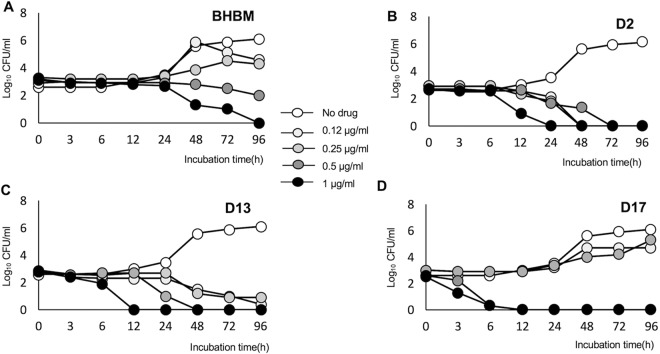
Killing activity of BHBM (A), D2 (B), D13 (C), and D17 (D). Killing activity was determined using an *in vitro* killing assay in which the compounds were coincubated with C. neoformans cells at 37°C, 5% CO_2_, pH 7.4. The number of CFU is counted during 96 h of incubation. All of the compounds displayed antifungal activity in a dose-dependent manner.

Nineteen derivatives of BHBM were purchased from ChemBridge and screened for activity against C. neoformans. Five compounds showed antifungal activity, ranging from a MIC_80_ of 0.06 μg/ml to 1 μg/ml (see Table S1 in the supplemental material). Out of the five, three compounds, called D2, D13, and D17, were selected for further studies because of their lower toxicity on A549 and HepG2 mammalian cell lines (Table 1). The three selected compounds and BHBM were resynthesized in our laboratory at high purity and used for further studies. D13 possessed an especially high selectivity index (>4,000) compared to the other 3 compounds. A time-kill assay was used to compare the killing activity of BHBM with that of the other three derivatives. All of the compounds were fungicidal and exhibited a dose-dependent killing of C. neoformans (Fig. 1). The derivatives killed the cells faster than BHBM. D17 killed in as little as 6 h, but it proved to be more toxic than the parent drug. D13 displayed a faster killing activity and, importantly, a very low toxicity in mammalian cells. For these reasons, D13 was selected to be tested in animal models of cryptococcosis, candidiasis, pulmonary aspergillosis, and pneumocystosis.

**TABLE 1:**
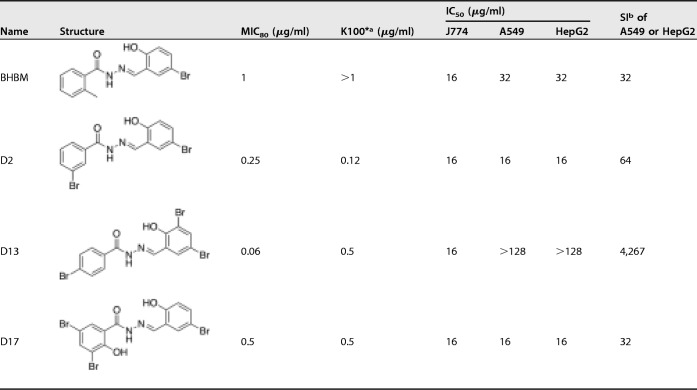
MIC_80_, *in vitro* killing activity, and cytotoxicity results of BHBM and three derivatives against C. neoformans

a K100* is the minimum concentration of the drug that kills 100% of C. neoformans cells in 48 h.

b The selectivity index (SI) is a ratio of IC_50_ against mammalian cell line and MIC_80_ against C. neoformans.

### Synergistic studies.

In order to assess if D13 had a synergistic effect with clinically used drugs, we performed a synergistic assay against different fungal strains (Table 2). D13 showed synergistic effect against C. neoformans when combined with existing antifungals. Interestingly, when combined with itraconazole, D13 showed a good synergistic activity (fractional inhibitory index [FIC] score of 0.37) against C. albicans clinical isolates resistant to fluconazole. Intriguingly, D13 was also able to resensitize to fluconazole a Candida krusei isolate clinically resistant to the drug. This effect was not observed when Candida krusei ATCC 6258 was used. When tested against A. fumigatus, D13 improved the performance of both voriconazole and itraconazole.

**TABLE 2:** Synergistic effect of D13 combined with commercially available drugs on different fungi

Fungal strain^*a*^	MIC (μg/ml) alone^*b*^						MIC (μg/ml) in combination					FIC index				
	D13	Flu	Vori	Itra	Caspo	AB	D13-Flu	D13-Vori	D13-Itra	D13-Caspo	D13-AB	D13-Flu	D13-Vori	D13-Itra	D13-Caspo	D13-AB
CA	>1	2.5	ND	0.06	0.12	ND	0.03/1.25	ND	0.25/0.03	0.125/0.06	ND	0.51	ND	0.62	0.56	ND
CA Flu-R	>1	>16	ND	2	0.06	ND	>1/>16	ND	0.25/0.5	0.25/0.03	ND	2	ND	0.37	0.62	ND
CK Flu R1	>1	16	ND	1	1	ND	0.25/1	ND	0.5/0.5	0.25/0.25	ND	0.31	ND	0.75	0.37	ND
CK Flu R2	>1	>16	ND	1	1	ND	>1/>16	ND	0.5/0.5	0.25/0.25	ND	2	ND	0.75	0.37	ND
CN	0.5	2.5	4	0.06	ND	0.25	0.25/0.125	0.125/1	0.06/0.015	ND	0.25/0.015	0.56	0.5	0.37	ND	0.56
AF	>1	>10	1	0.25	ND	ND	>1/>10	0.015/0.5	0.03/0.125	ND	ND	2	0.507	0.515	ND	ND

a CA, Candida albicans A39; CA Flu-R, Candida albicans 3022; CK Flu R1, Candida krusei; CK Flu R2, Candida krusei ATCC 6258; CN, Cryptococcus neoformans H99; AF, Aspergillus fumigatus 293.

b Flu, fluconazole; Vori, voriconazole; Itra, itraconazole; Caspo, capsofungin; AB, amphotericin B; ND, not determined.

### Antifungal activity against cryptococcosis.

**FIG 2:**
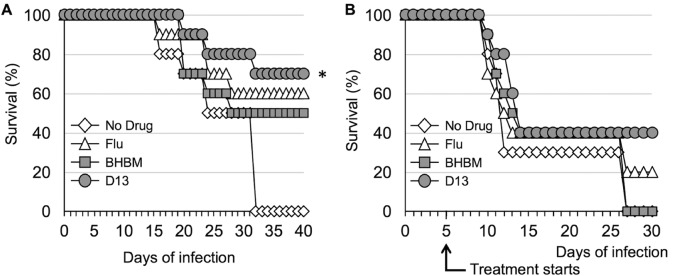
Survival studies of mice infected intranasally with 5 × 10^5^ C. neoformans cells and treated through i.p. injection. (A) Treatment started the day of infection with administration of 1.2 mg/kg/day. *, D13 versus no drug, *P* value of 0.0018. (B) Treatment started 5 days after infection with administration of 1.2 mg/kg/day.

BHBM and D13 were tested in a mouse model of cryptococcosis to assess their antifungal activity. In the first study, mice were treated intraperitoneally (i.p.) with BHBM or D13 at 1.2 mg/kg of body weight/day immediately after intranasal infection with 5 × 10^5^ C. neoformans cells. Fluconazole (Flu) at the same dose was used as a control. The mice that survived included 70% treated with D13, 50% treated with BHBM, and 60% treated with fluconazole for 40 days, whereas 100% of untreated mice died within 32 days (Fig. 2A) (*P* = 0.0018 for D13-treated mice versus untreated mice).

A second survival study was performed under the same conditions as those described above, except treatment started 5 days after the intranasal injection of fungal cells. Compared to the first study, untreated mice started dying earlier at about day 10. However, 40% of mice treated with D13 and 20% of mice treated with fluconazole survived until the end of the experiment. All untreated mice and the ones treated with BHBM died by day 30 (Fig. 2B).

**FIG 3:**
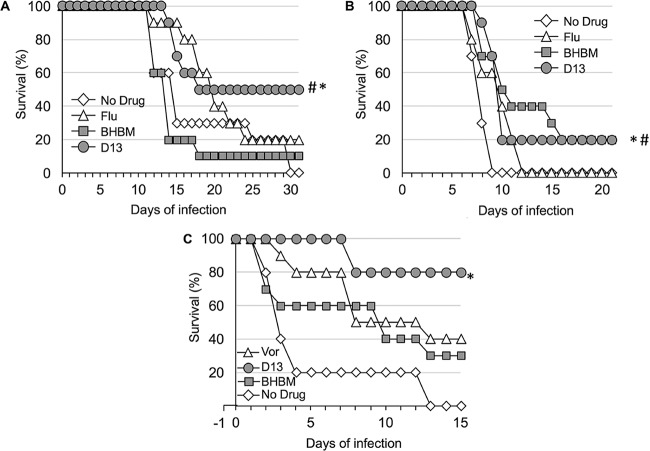
Effect of 20 mg/kg/day oral administration against cryptococcosis, candidiasis, and aspergillosis. (A) Survival of mice infected intranasally with 5 × 10^5^ C. neoformans cells and treated with fluconazole, BHBM, or D13. *, D13 versus no drug, *P* value of 0.0018; #, D3 versus BHBM, *P* value of 0.0057. (B) Survival of mice infected intravenously with 10^4^ C. albicans cells and treated with fluconazole, BHBM, or D13. *, D13 versus no drug, *P* value of 0.0004; #, BHBM versus no drug, *P* value of 0.015. (C) Survival of mice infected intranasally with 2 × 10^4^ conidia of A. fumigatus and treated with voriconazole, BHBM, or D13. *, D13 versus no drug, *P* value of <0.001.

A third survival study was carried out to evaluate the oral efficacy of the compounds against cryptococcosis. The mice were infected intranasally as described in the first study, followed by oral administration of D13, BHBM, or fluconazole at a dose of 20 mg/kg/day starting the day of infection. The mice that survived until the end of the experiment included 50% of those treated with D13, 10% of those treated with BHBM, and 20% of those treated with fluconazole, whereas all untreated mice died (Fig. 3A). There was a statistically significant difference between D13-treated and untreated or BHBM-treated mice, with a *P* value of 0.018 and 0.0057, respectively. At 32 days posttreatment, mice treated with fluconazole (2 mice) or D13 (5 mice) were sacrificed and the brains and lungs excised, and then the samples were examined for CFU. Fluconazole-treated mice showed numerous colonies in the lungs and brain, whereas only one D13-treated mouse showed fungal colonies and only in the lung (data not shown). All brains recovered from D13-treated mice were clear of fungal cells (data not shown).

### Antifungal activity against invasive candidiasis.

BHBM and D13 were tested for their efficacy in a mouse model of invasive candidiasis. The mice were infected intravenously with 10^4^ cells of C. albicans, and oral treatment through gavage was started as described above for the cryptococcosis model. After 21 days of infection, 20% of mice treated with BHBM and D13 survived, whereas fluconazole-treated or untreated mice died within 12 days (D13 versus no drug, *P* value of 0.0004; BHBM versus no drug, *P* value of 0.0015) (Fig. 3B).

### Antifungal activity against pulmonary aspergillosis.

We next tested BHBM and D13 in a mouse model of invasive pulmonary aspergillosis. The mice were immunosuppressed with glucocorticoid the day before intranasal infection with 2 × 10^4^ conidia from Aspergillus fumigatus, and the oral treatment was started the same day. Voriconazole was used instead of fluconazole as a control. After 15 days, 80% of D13-treated mice survived, whereas 40% of voriconazole-treated and 30% of BHBM-treated mice survived. All untreated mice died within 13 days (*P* value of <0.0001 for D13 versus no drug) (Fig. 3C). At the endpoint, the lungs of the surviving mice were excised and processed to assess fungal burdens. Even though the CFU quantification for A. fumigatus is not ideal, only one D13-treated mouse showed evidence of colonies, whereas all of the other D13-treated mice seemed to have cleared the infection (data not shown). All of the voriconazole- or BHBM-treated mice showed evidence of colonies after the lungs were assessed for CFU (data not shown). The reduction of fungal burden was also confirmed by histology (data not shown).

### Antifungal activity against Pneumocystosis.

The *in vivo* activity of D13 was tested in a murine model of pneumocystosis. Mice were treated with corticosteroids prior to infection to induce immunosuppression. Three groups of mice were included: (i) infected mice treated with vehicle as a negative control, (ii) infected mice treated with trimethoprim-sulfamethoxazole as a positive control, and (iii) infected mice treated with D13 intraperitoneally at a dose of 7.2 mg/kg/day. The mice treated with D13 did not show better survival and died at the same rate as the negative control. No differences were seen in the number of asci or nuclei in lung homogenates of the untreated versus the D13-treated mice (Fig. S1).

### Pharmacokinetic studies.

**FIG 4:**
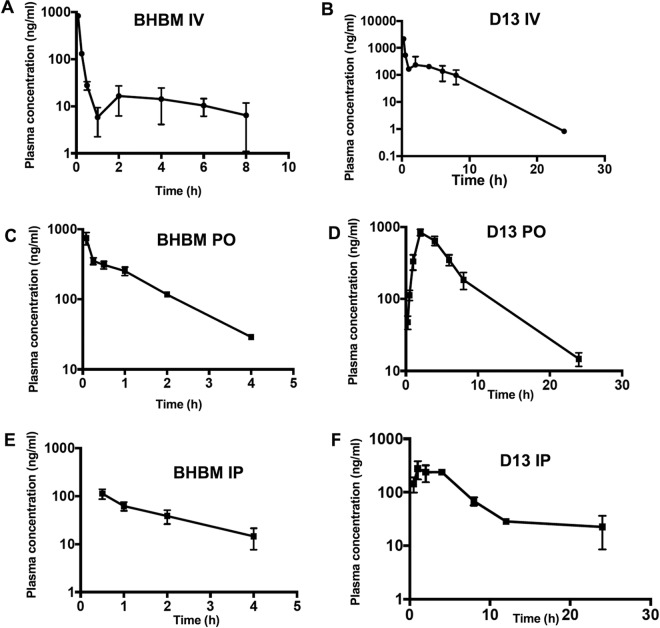
Pharmacokinetic studies of BHBM and D13. (A) BHBM administered i.v. at 1 mg/kg. (B) D13 administered i.v. at 1 mg/kg. (C) BHBM administered p.o. at 20 mg/kg. (D) D13 administered p.o. at 20 mg/kg. (E) BHBM administered i.p. at 1.6 mg/kg. (F) D13 administered i.p. at 1.6 mg/kg.

Pharmacokinetic studies were performed to assess the distribution of BHBM and D13 in the bloodstream. Plasma concentration was determined upon intraperitoneal (i.p.), intravenous (i.v.), or oral (p.o.) administration of the compounds. Upon i.p. administration, BHBM levels decreased rapidly and could not be detected after 4 h. D13 levels, however, increased initially, decreased gradually, and could still be detected after 20 h following i.p. injection. BHBM levels appeared to decrease immediately after i.v. injection but could be detected until 8 h in the plasma. D13 showed a more typical profile for oral administration and persisted at high concentration in the bloodstream for a longer time (Fig. 4).

### Metabolic stability studies.

The metabolic stability of BHBM and D13 was assessed using mouse and human liver microsomes. D13 possessed a longer half-life (*t*_1/2_) and slower clearance than BHBM in both human and mouse liver microsomes (Table 3). D13 was also found to be more stable than BHBM in the presence of the reducing enzyme NADPH. At the end of 60 min, nearly 60% of D13 remained in both human and mouse liver microsomes, whereas <1% BHBM remained in human liver microsomes and ∼44% remained in mouse liver microsomes in the presence of NADPH (Table 4).

**TABLE 3:** Metabolic stability of BHBM and D13 in human and mouse liver microsomes

Compound and species	*t*_1/2_ (min)	CL_int_^*a*^ (μl/min/mg protein)
Verapamil		
Human	12.19	113.72
Mouse	9.15	151.48
BHBM		
Human	48.81	28.40
Mouse	7.33	189.21
D13		
Human	103.09	13.45
Mouse	88.58	15.65

a CL_int_, intrinsic clearance.

**TABLE 4:** Metabolic stability of BHBM and D13 in human and mouse liver microsomes in the presence and absence of NADPH

Compound and species	Assay format	% remaining at:				
		0 min	15 min	30 min	45 min	60 min
Verapamil						
Human	With NADPH	100.00	28.59	11.61	5.43	3.23
	Without NADPH	100.00	99.39	100.00	95.15	93.33
Mouse	With NADPH	100.00	14.64	4.72	1.79	1.04
	Without NADPH	100.00	79.63	91.36	90.12	96.91
BHBM						
Human	With NADPH	100.00	83.24	62.53	51.28	43.93
	Without NADPH	100.00	100.96	88.46	81.25	82.69
Mouse	With NADPH	100.00	25.31	4.93	1.52	0.84
	Without NADPH	100.00	108.81	89.87	86.34	84.14
D13						
Human	With NADPH	100.00	83.88	73.00	73.10	64.95
	Without NADPH	100.00	96.49	86.55	81.87	88.89
Mouse	With NADPH	100.00	80.84	69.28	64.57	62.25
	Without NADPH	100.00	90.73	88.08	82.78	80.79

### Affinity toward hERG potassium channel.

In order to assess the affinity of our compounds toward the cardiac potassium channel, BHBM and D13 were tested for their inhibitory activity against the human ether-a-go-go-related gene (hERG) potassium channel. With a 50% inhibitory concentration (IC_50_) of 11.5 μM for BHBM and >30 μM for D13 (0.009 μM for dofetilide), neither compound displayed affinity.

### Caco-2 permeability.

The permeability of BHBM and D13 in the Caco-2 cell line was assessed. Both BHBM and D13 had an efflux ratio of <2. D13 possessed a lower permeability value (*P*_app_) than BHBM, which was less than 1 in both directions. BHBM had a higher *P*_app_ from A to B than from B to A. Nearly 40% BHBM was recovered in both directions, whereas the recovery for D13 was twice as high from basolateral to apical (BL→AP) as that from AP→BL compartments (Table 5).

**TABLE 5:** Caco-2 permeability of BHBM and D13^*a*^

Compound	*P*_app(A-B)_ (10^−6^, cm/s)	*P*_app(B-A)_ (10^−6^, cm/s)	Efflux ratio	Recovery (%)	
				AP→BL	BL→AP
Propranolol	34.38	19.50	0.57	90.69	95.53
Digoxin	0.61	20.25	33.17	82.73	95.58
BHBM	14.28	4.71	0.33	41.88	39.05
D13	0.13	0.22	1.74	49.82	106.98

a *P*_app(A-B)_, permeability value apical to basolateral; *P*_app(B-A)_, permeability value basolateral to apical.

### Permeability through the BBB.

To assess if the compounds could pass the blood-brain barrier (BBB), we used a commercially available kit (BBB kit MBT-24; Pharmaco Cell Company Ltd.). Both BHBM and D13 were able to pass through the BBB in the monkey brain model in the first 5 min, but their ability to pass through decreased after 15 min (Table 6). The *P*_app_ value for BHBM was similar to that of the positive control (caffeine) in the first 5 min but decreased as time progressed. The *P*_app_ value for D13 was lower than that for BHBM but was still able to pass in the first 5 min (Table 6). When tested in the rat model, they retained their ability to pass through the BBB.

**TABLE 6:** *P*_app_ of D13 and BHBM in monkey BBB kit

Drug	Time (min)	*P*_app_ (10^−6^ cm/s)
Cyclosporine	5	1
Cyclosporine	15	1
Cyclosporine	30	1
Caffeine	5	20
Caffeine	15	25
Caffeine	30	25
BHBM	5	22
BHBM	15	9
BHBM	30	9
D13	5	6.8
D13	15	2.2
D13	30	1.1

## DISCUSSION

In this study, we screened 19 derivatives of an acylhydrazone, called BHBM, that was previously found by our group to possess antifungal activity against several fungi (25). Out of the 19 derivatives, we selected 3 compounds (D2, D13, and D17) that possessed strong antifungal activity and low toxicity in mammalian cells for further studies. All compounds displayed fungicidal *in vitro* activity in a dose-dependent manner. In the *in vitro* killing assay, it was observed that the derivatives killed C. neoformans cells faster than BHBM at 1 μg/ml (Fig. 1). D13 in particular was highly active both *in vitro* and *in vivo* against C. neoformans.

D13 improved the efficacy of commercially available drugs when a synergistic assay was performed against different fungi. Very interestingly, we noticed that when D13 was used in combination with fluconazole in a C. krusei clinically resistant strain, the fluconazole efficacy was reestablished, although this was not observed with C. krusei ATCC 6258. Perhaps the C. krusei clinical isolate displays fluconazole-resistant mechanisms linked to target overexpression and/or pump drug efflux, mechanisms that D13 may be able to overcome by disrupting membrane integrity through the inhibition of GlcCer. Also, when in combination with voriconazole and itraconazole, D13 was strongly synergistic against A. fumigatus.

In the animal model, D13 was found to be more effective against cryptococcosis when the treatment started on the day of infection rather than 5 days postinfection (Fig. 2). Although D13 was not as efficacious *in vitro* against C. albicans or A. fumigatus as it was against C. neoformans, in the animal models D13 performed better than fluconazole against C. albicans and better than voriconazole against A. fumigatus (Fig. 3), suggesting that these molecules hold great promise for antifungal drug development.

The lack of efficacy against P. murina in the mouse model of pneumonia was in contrast to the activity observed for the other fungi and from previous *in vitro* activity. Possible explanations include a suboptimal dosing schedule or the lack of or poor expression of neutral glycosphingolipids in these fungal cells, since such biochemical assessments have not been conducted. Moreover, a hyphal stage for Pneumocystis has not been identified.

Pharmacokinetic analysis help to determine the concentration of the drug in circulation over a time course (31). D13 displayed better bioavailability than BHBM and also a typical profile for an orally administered compound. When administered intravenously (i.v.), the concentration of BHBM decreased sharply within the first hour to 10 ng/ml and remained at that concentration for about 8 h. On the other hand, the concentration of D13 decreased more gradually over a period of 4 h upon i.v. administration (Fig. 4).

Metabolic stability studies give us information, such as clearance, *t*_1/2_, and bioavailability, thus helping us determine the dose and frequency of administration for a drug molecule (32). D13 had lower clearance and lower *t*_1/2_ than BHBM in both human and mouse liver microsomes, thus explaining why the antifungal activity is retained for a longer period of time in our animal model. BHBM seemed to be highly metabolically labile in the presence of NADPH in mouse liver microsomes. Since most of the preclinical studies are done in murine models, it is essential to have a molecule that is stable in mouse liver microsomes as well. D13 was more stable than BHBM in both human and mouse liver microsomes in the presence and absence of NADPH.

hERG codes for a potassium channel that is essential for cardiac repolarization (33). A wide range of drugs block the hERG channel, resulting in cardiac arrhythmia (34). Thus, it is highly essential to screen compounds for cardiac safety in early stages of drug discovery (35). BHBM and D13 were tested for their affinity toward the hERG channel in a patch clamp assay. BHBM had an IC_50_ of 11.5 μM, displaying moderate affinity toward the hERG channel, and D13 had an IC_50_ of >30 μM, displaying low affinity. These results suggest that BHBM and D13 are not cardiotoxic.

Caco-2 cells are a cancer cell line from the human colon epithelium and are used as a model to assess intestinal absorption of drugs (36). The efflux ratio of both BHBM and D13 was <2, suggesting neither compound was a substrate for efflux pumps such as P-gp and, thus, were transported across the membrane by passive diffusion. The percent recovery for BHBM was ∼40% in both directions, whereas the recovery for D13 was twice as high from BL→AP as that from AP→BL, although neither BHBM nor D13 is metabolized by Caco-2 cells. These results suggest D13 is more suitable than BHBM for oral administration.

The BBB prevents the passage of most of the drugs from the blood to the brain (37). Because most invasive fungal infections eventually affect the brain tissue, having a drug that can kill the fungi not only in the lungs but also in the brain is essential (38). The assay was done using rat and monkey brain models. In the rat model both compounds passed through the BBB in a time-independent manner. In the monkey model both compounds passed within the first 5 min; however, their ability to pass through the BBB reduced with time (Table 6). These results suggest that this class of molecules penetrates the BBB and reaches the brain tissue.

Compared to BHBM, D13 was not only more fungicidal but also less toxic. D13 showed better survival of mice infected with C. neoformans and particularly with A. fumigatus than did BHBM. Promisingly, D13 seems to perform better than clinically recommended antifungal drugs such as fluconazole or voriconazole. Our studies confirmed that this new class of antifungal agents (acylhydrazones) targeting the sphingolipid pathway could be used as promising new lead molecules in the research and development of more efficacious and less toxic antifungal drugs.

## MATERIALS AND METHODS

### Strains, media, and reagents.

A series of clinical isolates and reference strains were used in this study, including Cryptococcus neoformans (strain H99), Candida albicans (strain A39), Aspergillus fumigatus (strain Af239), and Pneumocystis murina. The strains were obtained from existing collections at M. Del Poeta's laboratory (Stony Brook University), M. Cushion's laboratory (University of Cincinnati), and R. A. Cramer, Jr.’s laboratory (Dartmouth University). Yeast-peptone-dextrose (YPD), yeast nitrogen base (YNB), RPMI 1640, and Dulbecco's modified Eagle's medium (DMEM) were purchased from Invitrogen Life Technologies (Grand Island, NY). Fluconazole was purchased from Sigma-Aldrich (St. Louis, MO). Voriconazole was obtained from Pfizer (Rey Brook, NY).

### *In vitro* susceptibility testing.

MICs were determined by following the methods of the Clinical and Laboratory Standards Institute (CLSI), with modifications. YNB medium (pH 7.0, 0.2% glucose) buffered with HEPES was used for MIC studies. HEPES was used instead of morpholinepropanesulfonic acid (MOPS), because MOPS was found to inhibit the activity of this kind of compound. The compounds were serially diluted from 32 to 0.03 μg/ml in a 96-well plate. The inoculum was prepared as described in CLSI protocol M27A3 guidelines (39). The plates were incubated at 37°C with 5% CO_2_ for 24 to 72 h, and the optical density was measured at 450 nm. The MICs were determined as the lowest concentration of the compound that inhibited 80% of growth compared to the control.

### *In vitro* toxicity.

The murine macrophage cell line J774 and human cancer cell lines A549 and HepG2 were maintained in DMEM containing 10% fetal bovine serum (FBS) and 1% penicillin-streptomycin. At passage 7, 10^5^ cells/well in DMEM containing 10% FBS were transferred into 96-well plates and cultured for 14 h for the cells to adhere to the wells. The compounds were added to the cells at concentrations ranging from 0.03 to 128 μg/ml. The wells without the compound served as controls. The plate was incubated at 37°C with 5% CO_2_. After 24 or 48 h, the supernatant was removed and 50 μl of 5-mg/ml 3-(4,5-dimethylthiazol-2-yl)-2,5-diphenyltetrazoliumbromide (MTT) solution in phosphate-buffered saline (PBS) was added to each well. The plates were incubated for an additional 4 h. The formazan crystal formed inside the cell was dissolved by adding 50 μl of dimethyl sulfoxide (DMSO). The optical density was measured at 570 nm.

### *In vitro* killing assay.

C. neoformans cells from a culture grown overnight were washed in PBS and resuspended in YNB buffered with HEPES at pH 7.4. The cells were counted, and 2 × 10^4^ cells were incubated with different concentrations of the drugs in a final volume of 10 ml with a final concentration of 0.5% DMSO. The tubes were then incubated at 37°C with 5% CO_2_ on a rotary shaker at 200 rpm. Aliquots were taken at the time points indicated and diluted, and 100-µl portions were plated onto YPD plates. YPD plates were incubated in a 30°C incubator, and after 48 h the numbers of CFU were counted and recorded.

### Synergistic assay.

Synergistic activity was assayed by calculating the fractional inhibitory index (FIC). Briefly, in a 96-well plate, drug A (D13) was serially diluted from 16 to 0.015 μg/ml (11 dilutions), whereas drug B (either fluconazole, amphotericin B, caspofungin, itraconazole, or voriconazole) was serially diluted from 12 to 0.19 μg/ml for fluconazole, 5 to 0.078 μg/ml for amphotericin B, or 8 to 0.007 μg/ml for caspofungin, itraconazole, or voriconazole (seven dilutions). The FIC was defined as (MIC combined/MIC drug A alone) + (MIC combined/MIC drug B alone). Synergism was categorized with the following scale: strongly synergistic effect, FIC of <0.5; synergistic effect, FIC of <1; additive effect, FIC of 1; no effect, FIC between 1 and 2; antagonistic effect, FIC of >2.

### Animal study for cryptococcosis.

For survival studies, 4-week-old CBA/J (Envigo) male mice were used. They were divided into sets of 10 mice for each treatment or control group. Mice were infected intranasally with 20 μl of a suspension containing 5 × 10^5^ C. neoformans cells and subsequently treated through intraperitoneal (i.p.) injection with 1.2 mg/kg/day of BHBM, D13, or fluconazole (as a drug control) in a final volume of 100 μl of water containing 0.5% DMSO. The untreated control group mice received 100 μl of water containing 0.5% DMSO. A second survival experiment was performed as described above, but the treatment was started 5 days after the infection. A third survival study was performed using the same design, but the mice were treated starting the day of infection with 20 mg/kg/day of BHBM, D13, or fluconazole in a final volume of 100 μl of 30% polyethylene glycol (PEG) in a saline buffer. The untreated control group mice received 100 μl of 30% PEG in a saline buffer. Gavage was used as the route of administration.

### Animal study for candidiasis.

For survival study, 8-week-old CBA/J (Envigo) male mice were used. They were divided into sets of 10 mice for each treatment or control group. Mice were infected intravenously with 100 μl of a suspension containing 10^5^ cells of C. albicans strain A39 and subsequently treated through gavage with 20 mg/kg/day of BHBM, D13, or fluconazole (as a drug control) in a final volume of 100 μl of 30% PEG in saline buffer. The untreated control group mice received 100 μl of 30% PEG in saline buffer. Mice were fed ad libitum, monitored every day for signs of discomfort, and sacrificed subsequently.

### Animal study for aspergillosis.

For survival study, 8-week-old CBA/J (Envigo) male mice were used. They were divided into sets of 10 mice for each treatment or control group. The day prior to infection, the mice were immunosuppressed by using triamcinolone acetonide (1 mg/mouse) subcutaneously (40). On day 0, mice were infected intranasally with 20 μl of a suspension containing 2 × 10^4^ conidia of A. fumigatus strain Af293 and were treated through gavage with 20 mg/kg/day of BHBM, D13, or voriconazole (as a drug control) in a final volume of 100 μl of 30% PEG in saline buffer. The untreated control group mice received 100 μl of 30% PEG in saline buffer. Mice were fed ad libitum and were given 50% grapefruit juice in place of normal drinking water to avoid the liver metabolism of the drugs (41). Mice were monitored every day for discomfort and sacrificed subsequently.

### Animal study for pneumocystosis.

C3H/HeN mice ordered from the National Cancer Institute were infected with P. murina pneumonia through exposure to mice with a fulminant P. murina infection (seed mice). These mice are immune suppressed by the addition of dexamethasone at 4 mg/liter to the drinking water. Sulfuric acid at 1 ml/liter was also added to the drinking water for disinfection. The seed mice were rotated within the cages for 2 weeks and then removed. After the mice had developed a moderate infection level (approximately 5 weeks), the mice were divided into a negative-control group (control steroid), positive-control group (trimethoprim-sulfamethoxazole), and D13 group. Compound was solubilized in DMSO for a stock solution of 50 mg/ml. Compound was refrigerated before and after solubilization. Stock solution was diluted in PBS to a final concentration of 1.8 mg/ml. Mice were intraperitoneally (i.p.) injected with 100 μl of diluted stock for a total dose of approximately 180 μg/day or 7.2 mg/kg/day based on average mouse weights of 25 g. At the end of the treatment, the mice were euthanized by CO_2_ and lungs were processed for analysis. Slides were made from the lung homogenates at different dilutions and stained with Diff-Quik to quantify the trophic forms and cresyl echt violet to quantify the asci. Efficacy is based on the reduction of organism burden between the treatment groups and the negative-control group as determined by microscopic evaluation.

### Statistics.

All data are expressed as means ± standard deviations. No samples or animals were excluded from the analysis. For animal studies, group sizes were chosen when sufficient to reach a statistical power of at least 80% (https://www.statisticalsolutions.net/pssZtest_calc.php). Mice were assigned randomly to treatment groups, and both males and females were used. Statistical analysis for survival studies was performed using Kruskal-Wallis test. Statistical analysis for tissue burden, trophic form, and asci counts was performed using analysis of variance (ANOVA). Data met the assumption of a normal distribution as determined by statistical software, and variance was similar between groups that were statistically compared. Statistical tests were carried out using GraphPad Prism (La Jolla, CA, USA) v. 400 software for Mac. Replicates used were biological replicates. Results were considered significant at a *P* value of ≤0.05.

### Animal study approval.

Mice were fed ad libitum and monitored every day for discomfort and signs of disease. Mice showing weight loss, lethargy, tremor, or inability to reach food or water were euthanized, and survival was counted until that day. Euthanasia was performed with CO_2_ asphyxiation with 100% FiCO_2_ for 2 min, followed by cervical dislocation. Mouse experiments were performed in full compliance with a protocol approved by Stony Brook University (study number 341888; IACUC number 2012-1967) and the University of Cincinnati Institutional Animal Care and Use Committee (ACORP number 14-01-14-01) and in compliance with the United States Animal Welfare Act (Public Law 98-198). The experiments were carried out in facilities accredited by the Association for Assessment and Accreditation of Laboratory Animal Care.

### Pharmacokinetic studies.

For i.p. study, BHBM and D13 were dissolved in a mixture of cremophore-ethanol (1:1) to prepare a 10-mg/ml stock solution. The stock solution was diluted in PBS to obtain 200-μg/ml and 400-μg/ml solutions for i.p. administrations in C3H/HeN mice (*n* = 3). The compounds were administered to control (healthy) mice or immunocompromised mice infected with P. murina at doses of 0.8 mg/kg and 1.6 mg/kg via tail vein injection or intraperitoneal injection in a final volume of 100 μl. The mice were sacrificed and blood samples were collected predose and 0.5, 1, 2, 4, 8, 12, and 24 h after administration into K_2_EDTA-containing tubes. The samples were centrifuged immediately, and plasma was collected and stored at −80°C until bioanalysis. Plasma samples were extracted using methylene chloride.

Briefly, 50 μl of the plasma sample was taken into a glass vial, and 10 μl of internal standard *N*′-(3-bromobenzylidene)-4-hydroxybenzohydrazide was added. After the contents of the glass vial were mixed, 1 ml of methylene chloride was added to the vial and the samples were vortex mixed for 30 s, followed by centrifugation for 5 min. Eight hundred microliters of supernatant then was transferred to another tube and evaporated to dryness using a centrifugal evaporator. The residue was reconstituted in 100 μl acetonitrile-water (50:50) solution, mixed, and transferred to mass spectrometry vials. Separation was performed under isocratic reverse-phase chromatographic conditions using a Waters XBridge C_18_ column (3.5 μm; 2.1 by 100 mm) (Waters, Milford, MA), a Finnigan Surveyor MS pump (Thermo Fisher Scientific), and a Finnigan Micro AS autosampler (Thermo Fisher Scientific).

The mobile phase consisted of water-acetonitrile with 0.1% formic acid (50:50) run at a flow rate of 200 μl/min. Five-microliter aliquots then were analyzed using an LTQ-FT liquid chromatography-tandem mass spectrometer (LC-MS/MS) with the electrospray source in the positive ion mode (Thermo Fisher Scientific). For i.v. and p.o. administration, the formulation consisted of a 0.5-mg/ml solution of PEG 400-DMA (9:1). A volume of 1.21 mg of D13 was dissolved in 0.242 ml of dimethacrylate (DMA) by vortexing and sonication, and then 2.178 ml of PEG440 was added with vortexing and sonication until a clear solution was obtained. For a 4-mg/ml solution of PEG 400-DMA (9:1), 2.13 mg of D13 was dissolved in 0.053 ml of DMA by vortexing and sonication, and then 0.479 ml of PEG 440 was added with vortexing and sonication until a clear solution was obtained. For i.v. and p.o. administration of BHBM, the formulation consisted of a 0.5-mg/ml solution of 5% DMSO and 5% solutol in water. A volume of 0.98 mg of BHBM was dissolved in 0.098 ml of DMSO with vortexing and sonication. A volume of 0.098 ml of solutol was added with vortexing and sonication, and then 1.764 ml of water was added with vortexing and sonication until a clear solution was achieved. For the 2-mg/ml solution of 5% DMSO and 5% solutol in water, 2.33 mg of BHBM was dissolved in 0.058 ml of DMSO with vortexing and sonication. A volume of 0.058 ml of solutol was used for vortexing and sonication, and then 1.049 ml of water was added with vortexing and sonication until a clear solution was achieved.

### Metabolic stability studies.

To test for metabolic stability, 40 μl of 10 mM NADPH solution was added to each well of a 96-well plate. The final concentration of NADPH was 1 mM. The mixture was prewarmed at 37°C for 5 min. The negative-control samples were prepared by replacing NADPH solutions with 40 μl of ultrapure H_2_O. The negative control was used to exclude the misleading factor that resulted from the instability of the chemical itself. Samples with NADPH were prepared in duplicate. Negative controls were prepared in singlet. The reaction was started with the addition of 4 μl of 200 μM control compound or test compound solutions. Verapamil was used as a positive control in this study. The final concentration of test compound or control compound was 2 μM. Aliquots of 50 μl were taken from the reaction solution at 0, 15, 30, 45, and 60 min. The reaction was stopped by the addition of 4 volumes of cold acetonitrile with an internal standard (IS; 100 nM alprazolam, 200 nM imipramine, 200 nM labetalol, and 2 μM ketoprofen). Samples were centrifuged at 3,220 × *g* for 40 min. An aliquot of 90 μl of the supernatant was mixed with 90 μl of ultrapure H_2_O and then used for LC-MS/MS analysis.

### Caco-2 permeability studies.

The Caco-2 permeability studies were performed as follows. (i) For the preparation of Caco cells, 50-μl and 25-ml volumes of cell culture medium were added to each well of the Transwell insert and reservoir, respectively. The HTS Transwell plates then were incubated at 37°C, 5% CO_2_ for 1 h before cell seeding. Caco-2 cells were diluted to 6.86 × 10^5^ cells/ml with culture medium, and 50-μl volumes of cell suspension were dispensed into the filter well of the 96-well HTS Transwell plate. Cells were cultivated for 14 to 18 days in a cell culture incubator at 37°C, 5% CO_2_, 95% relative humidity. Cell culture medium was replaced every other day, beginning no later than 24 h after initial plating. (ii) For the preparation of stock solutions, 10 mM stock solutions of test compounds were prepared in DMSO. The stock solutions of positive controls were prepared in DMSO at a concentration of 10 mM. Digoxin and propranolol were used as control compounds in this assay. (iii) For the assessment of cell monolayer integrity, medium was removed from the reservoir and each Transwell insert and replaced with prewarmed fresh culture medium. Transepithelial electrical resistance (TEER) across the monolayer was measured using a Millicell epithelial volt-Ohm measuring system (Millipore, USA). The plate was returned to the incubator once the measurement was done. The TEER value was calculated according to the following equation: TEER measurement (ohms) × area of membrane (cm^2^) = TEER value (ohm · cm^2^). The TEER value should be greater than 230 Ω · cm^2^, which indicates a well-qualified Caco-2 monolayer. (iv) The assay was performed as follows. The Caco-2 plate was removed from the incubator and washed twice with prewarmed Hanks' balanced salt solution (HBSS; with 10 mM HEPES, pH 7.4) and then incubated at 37°C for 30 min. The stock solutions of control compounds were diluted in DMSO to get 1 mM solutions and then diluted with HBSS (10 mM HEPES, pH 7.4) to get 5 μM working solutions. The stock solutions of the test compounds were diluted in DMSO to get 1 mM solutions and then diluted with HBSS (10 mM HEPES, pH 7.4) to get 5 μM working solutions. The final concentration of DMSO in the incubation system was 0.5%. To determine the rate of drug transport in the apical to basolateral direction, 75 μl of 5 μM working solution of test compound was added to the Transwell insert (apical compartment), and the wells in the receiver plate (basolateral compartment) were filled with 235 μl of HBSS (10 mM HEPES, pH 7.4). To determine the rate of drug transport in the basolateral to apical direction, 235 μl of 5 μM working solution of test compound was added to the receiver plate wells (basolateral compartment), and then the Transwell inserts (apical compartment) were filled with 75 μl of HBSS (10 mM HEPES, pH 7.4). Time zero samples were prepared by transferring 50 μl of 5 μM working solution to wells of the 96-deep-well plate, followed by the addition of 200 μl cold acetonitrile or methanol containing appropriate IS. The plates were incubated at 37°C for 2 h. At the end of the incubation, 50-μl samples from donor sides (apical compartment for AP→BL flux and basolateral compartment for BL→AP) and receiver sides (basolateral compartment for AP→BL flux and apical compartment for BL→AP) were transferred to wells of a new 96-well plate, followed by the addition of 4 volumes of cold acetonitrile or methanol containing appropriate IS. Samples were vortexed for 5 min and then centrifuged at 3,220 × *g* for 40 min. An aliquot of 100 μl of the supernatant was mixed with an appropriate volume of ultrapure water before LC-MS/MS analysis. To determine the Lucifer yellow leakage after a 2-h transport period, a stock solution of Lucifer yellow was prepared in ultrapure water and diluted with HBSS (10 mM HEPES, pH 7.4) to reach a final concentration of 100 μM. One hundred microliters of the Lucifer yellow solution was added to each Transwell insert (apical compartment), followed by filling the wells in the receiver plate (basolateral compartment) with 300 μl of HBSS (10 mM HEPES, pH 7.4). The plates were incubated at 37°C for 30 min, and 80-μl samples were removed directly from the apical and basolateral wells (using the basolateral access holes) and transferred to wells of new 96-well plates. The Lucifer yellow fluorescence signal (to monitor monolayer integrity) was measured in a fluorescence plate reader at 485-nm excitation and 530-nm emission.

### hERG affinity.

The patch clamp assay was performed according to the following instructions. The coverslip was removed from the cell culture dish and placed on the microscope stage in a bath chamber. A desirable cell was located using the 10× objective. The tip of the electrode was located under the microscope using the 10× objective by focusing above the plane of the cells. Once the tip was in focus, the electrode was advanced downwards toward the cell using the coarse controls of the manipulator while simultaneously moving the objective to keep the tip in focus. When directly over the cell, the 40× objective was used with fine controls of the manipulator to approach the surface of the cell in small steps. Gentle suction was applied through the side port of the electrode holder to form a giga-Ohm seal. Cfast was used to remove the capacity current that is in coincidence with the voltage step. The whole-cell configuration was obtained by applying repetitive, brief, strong suction until the membrane patch has ruptured. At this point set membrane potential to was set to −60 mV to ensure that hERG channels are not open. The spikes of capacity current then were cancelled using Cslow on the amplifier. Holding potential was set to to −90 mV for 900 ms, and current was recorded at 50 kHz and filtered at 10 kHz. Leaking current was tested at −80 mV for 500 ms. The hERG current was elicited by depolarizing at +30 mV for 4.8 s, and then the voltage was taken back to −50 mV for 5.2 s to remove the inactivation and observe the deactivating tail current. The maximum amount of tail current size was used to determine hERG current amplitude. Current was recorded for 120 s to assess current stability. Only stable cells with recording parameters above the threshold were applied for the drug administrations. First, vehicle control was applied to the cells to establish the baseline. After allowing the current to stabilize for 3 min, compound was applied. hERG current in the presence of test compound was recorded for approximately 5 min to reach steady state, and then 5 sweeps were captured. For dose-response testing, compound was applied to the cells cumulatively from low to high concentrations. The positive-control dofetilide was used in this experiment to test the same batch of cells to ensure good performance of the cells and operations.

### Permeability through the blood-brain barrier.

A commercially available kit was used to examine permeability through the blood-brain barrier (BBB kit MBT-24; Pharmaco Cell Co. Ltd.). This kit allows us to perform a permeability assay using an *in vitro* reproduction of the blood-brain barrier using monkey brain. Briefly, in a 12-well plate there is a triple culture, with endothelial cells in the luminal part and perycites and astrocytes in the abluminal side connected by tight junctions. The luminal side of the well represents the blood side, whereas the abluminal side represents the brain side. The integrity of the blood-brain barrier is assessed by the measurement of TEER, which indicates the formation of tight junctions. To have an efficient system, the TEER should be >150 Ω cm^2^. The kit is kept frozen at −80°C until it is ready to use. Once thawed, it is activated by adding medium and changing it after 3 h and again after 24 h. After that it takes 3 days for the cells to adjust, and then the plate can be used in 4 days for the experiment. For drug testing, after assessing the TEER, the drugs were added in the chosen medium at the desired concentrations and at different time points. At the end of each time point the TEER was measured to assess if the BBB was still intact. After that the medium was collected and subjected to MS to measure the concentration of the samples in the brain side and calculate the permeability value (*P*_app_). If the *P*_app_ is greater than 20 it is very good, between 10 and 20 is good, between 2 and 10 it can pass through but in extremely small amounts, and values lower than 2 indicate the compound cannot pass. BHBM and D13 were tested alongside caffeine as a positive control and cyclosporine as a negative control.

## Supplementary Material

Supplemental material
