# Supplementary material for: Acylhydrazones as Antifungal Agents Targeting the Synthesis of Fungal Sphingolipids
Source: Antimicrob Agents Chemother. 2018 Apr 26;62(5):e00156-18. doi: 10.1128/AAC.00156-18 (PMC5923120; doi:10.1128/AAC.00156-18)
Supplement: Supplemental material [file supp_62_5_e00156-18__index.html]

Supplemental material 

# Acylhydrazones as Antifungal Agents Targeting the Synthesis of Fungal Sphingolipids

## Supplemental material

- Supplemental file 1 -

  Table S1 and Fig. S1

  PDF, 277K
