# Supplementary material for: Acylhydrazones as Antifungal Agents Targeting the Synthesis of Fungal Sphingolipids
Source: Antimicrob Agents Chemother. 2018 Apr 26;62(5):e00156-18. doi: 10.1128/AAC.00156-18 (PMC5923120; doi:10.1128/AAC.00156-18)
Supplement: Supplemental material [file AAC.00156-18_zac005187132s1.pdf]

Acylhydrazones as antifungal agents targeting the synthesis of fungal sphingolipids

Cristina Lazzarini[a,b,*], Krupanandan Haranahalli[c,*], Robert Rieger[d], Hari Krishna Ananthula[e], Pankaj B. Desai[e], Alan Ashbaugh[f], Michael J. Linke[f,g], Melanie T. Cushion[f,g], Bela Ruzsicska[c], John Haley[c,d], Iwao Ojima[c,h], and Maurizio Del Poeta[a,b,c,i,#]

[a]Department of Molecular Genetics and Microbiology, Stony Brook University, Stony Brook, New York, USA;

[b]Veterans Administration Medical Center, Northport, NY, USA;

[c]Institute of Chemical Biology and Drug Discovery, Stony Brook University, Stony Brook, New York, USA;

[d]Proteomics Center, Stony Brook University, New York, USA;

[e]Department of Pharmaceutical Sciences, University of Cincinnati, Cincinnati, Ohio, USA;

[f]Department of Veterans Affairs Medical Center, Cincinnati, Ohio, USA;

[g]University of Cincinnati College of Medicine, Cincinnati, Ohio, USA;

[h]Department of Chemistry, Stony Brook University, Stony Brook, New York, USA;

[i]Division of Infectious Diseases, School of Medicine, Stony Brook University, New York, USA

*Contributed equally

Running Title: Acylhydrazones as antifungal agents

# Corresponding Author:
Maurizio Del Poeta, M.D.
Department of Molecular Genetics and Microbiology
Division of Infectious Diseases
Stony Brook University
150 Life Science Building
Stony Brook, NY 11794
Tel: (631) 632-4024
Fax: (631) 632-9797
maurizio.delpoeta@stonybrook.edu

Supplementary Table 1: Structures and MIC_{80} of ChemBridge compounds.

Name	Structure	MIC_{80} (µg/mL)	Name	Structure	MIC_{80} (µg/mL)
BHBM	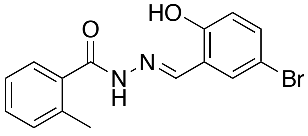	1	D10	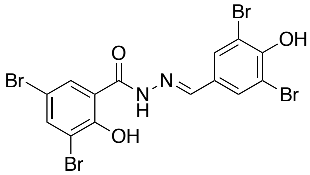	>16
D1	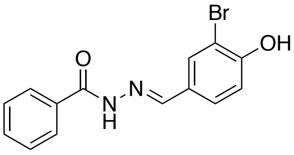	16	D11	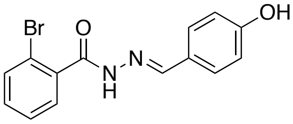	>16
D2	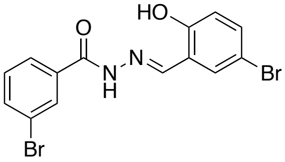	0.25	D12	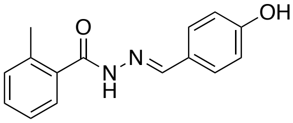	>16
D3	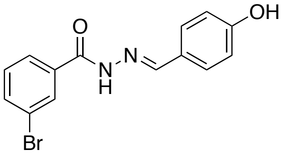	>16	D13	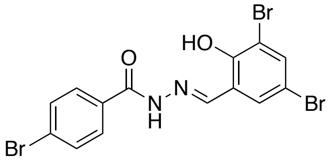	0.06
D4	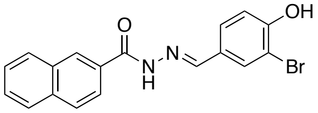	>16	D14	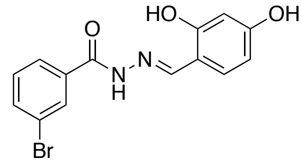	>16
D5	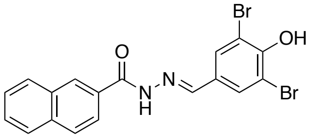	>16	D15	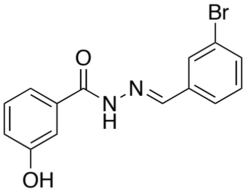	>16
D6	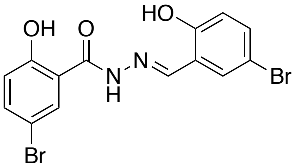	0.12	D16	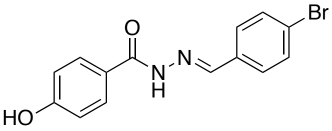	>16
D7	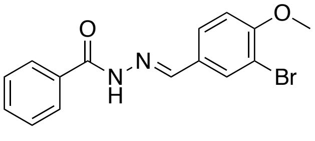	>16	D17	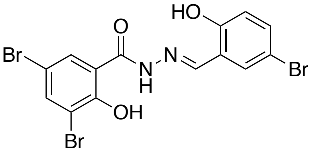	0.5

D8	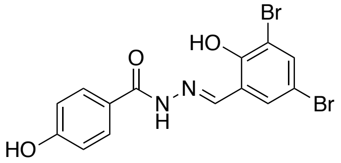	>16	D18	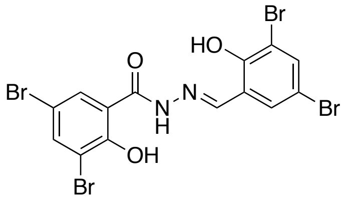	2
D9	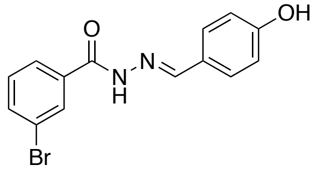	>16	D19	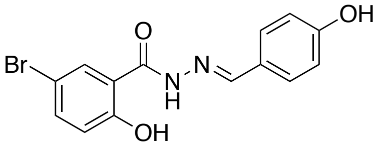	>16

Supplementary Figure 1

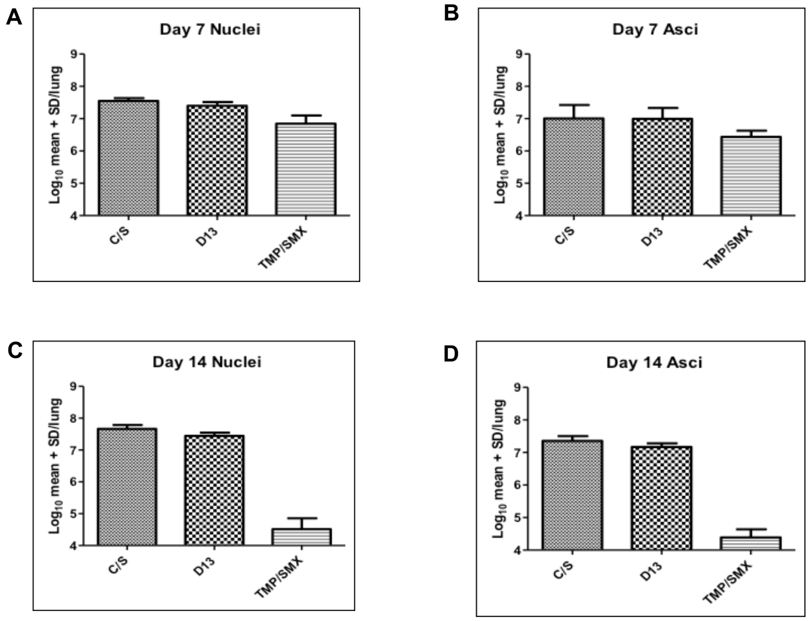

Supplementary Figure 1: Mean counts of asci and nuclei at Day 7 (A-B) and Day 14 (C-D) of infection of *P. murina* in the lung upon D13 or trimethoprim-sulfamethoxazole (TMP/SMX) intraperitoneal treatment using a corticosteroid-immunosuppressed mouse. C/S, vehicle (negative control).
